# The Prognostic Value of Amplification of the *MYCC* and *MYCN* Oncogenes in Russian Patients with Medulloblastoma

**DOI:** 10.3390/diseases13080238

**Published:** 2025-07-27

**Authors:** Alexander Chernov, Ekaterina Batotsyrenova, Sergey Zheregelya, Sarng Pyurveev, Vadim Kashuro, Dmitry Ivanov, Elvira Galimova

**Affiliations:** 1 Federal State Budgetary Educational Institution of Higher Education, “Saint Petersburg State Pediatric Medical University of the Ministry of Health of Russia”,194100 Saint Petersburg, Russia; bkaterina2009@yandex.ru (E.B.); sgerege-ly@bk.ru (S.Z.); dr.purveev@gmail.com (S.P.); kashuro@yandex.ru (V.K.); doivanov@yandex.ru (D.I.); 2 Federal State Budgetary Institution of Science “Institute of Experimental Medicine”, 197022 Saint Petersburg, Russia; 3 Department of Maxillofacial Surgery and Surgical Dentistry, Medical Institute of Saint Petersburg State University, 199034 Saint Petersburg, Russia; 4 Department of Anatomy and Physiology of Humans and Animals, Herzen State Pedagogical University of Russia, 191186 Saint Petersburg, Russia; 5 Sechenov Institute of Evolutionary Physiology and Biochemistry of the Russian Academy of Sciences, 197341 Saint Petersburg, Russia

**Keywords:** medulloblastoma, pediatric brain tumor, *MYCC*, MYCN amplification, 17q *gain*, 17p, 17p *del*, fluorescent in situ hybridization, the prognostic value of overall survival

## Abstract

**Background.** Medulloblastoma (MB) prognosis and response to therapy depend largely on genetic changes in tumor cells. Many genes and chromosomal abnormalities have been identified as prognostic factors, including amplification of *MYC* oncogenes, gains in 1q and 17q, deletions in 10q and 21p, or isochromosomes 17 (i(17)(q10)). The frequency of these abnormalities varies greatly between ethnic populations, but the frequency of specific abnormalities, such as *MYCC* and *MYCN* amplification, 17q gain, and deletions, in the Russian population is unknown. **Objective:** The aim is to study the frequency of *MYCC* and *MYCN* amplifications, 17q gain, and 17p deletion and determine their prognostic value in Russian patients with MB. **Methods.** This study was performed on MB cells obtained from 18 patients (12 boys and 6 girls, aged between 3 months and 17 years, with a median age of 6.5 years). Determination of cytogenetic aberrations was carried out using FISH assays with MYCC-SO, MYCN-SO, and MYCN-SG/cen2 probes, as well as cen7/p53 dual color probes and PML/RARα dual color probes (Abbott Molecular, USA). One-way ANOVA and Fisher’s F-test were used to compare the two groups. The differences were considered significant when *p* < 0.05. **Results.** In 77.7% of patients (14/18), the classical type of MB was present; in 16.7% (3/18), desmoplastic type; and in 5.6% (1/18), nodular desmoplasic types of neoplasms. Amplification of *MYC* genes was detected in 22.2% of Russian patients (*n* = 4 out of 18). Patients with *MYC* amplification had the worst overall survival (OS: 0% vs. 68%, *p* = 0.0004). Changes on the 17th chromosome were found in 58.3% of patients. Deletion of 17p occurred in 23.1%, and gain of 17q occurred in 46.2%. There were no significant differences in OS, clinical signs, or the presence of additional 17q material or 17p deletion among patients with MB. **Conclusions:** Amplification of the *MYC* gene is a predictor of poor overall survival to therapy and a high risk of metastatic relapse. This allows us to more accurately stratify patients into risk groups in order to determine the intensity and duration of therapy.

## 1. Introduction

Cancer is currently one of the most common and fatal diseases among humans. The main treatments for cancer include surgery, radiation therapy and chemotherapy [1]. However, radiotherapy and chemotherapy have low specificity, leading to damage to both tumor and healthy tissue, resulting in a low survival rate for treated patients due to drug resistance, tumor relapse, and metastasis. Recently, new targeted immunotherapy methods have been developed, including antibody-drug conjugates (ADCs), chimeric antigen receptor (CAR)-T therapy, and bispecific T-cell engaged therapy (BiTE) [1].

The most common (15.0–20.0%) malignant brain tumor in children is medulloblastoma (MB) [2]. Currently, there is increasing evidence that prognosis and response to therapy depend largely on the molecular and genetic features of tumor cells. According to WHO recommendations, at least four different histological types of malignant brain tumors (MBs) are recognized. Based on morphological criteria for histologic classification, these MBs can be divided into four categories: classic (CLA), desmoplastic-nodular (DN), malignant with extensive nodularity (MBE), and large-cell anaplastic (LCA) [3].

MBs are localized in the cerebellum and arise from neural progenitor stem cells (NSCs) [4]. These cells represent a small population of slowly growing cells that accumulate mutations and are resistant to treatment. If therapy has a damaging effect on a tumor, NSCs begin to proliferate and differentiate into other types of cancer cells, creating cellular and molecular diversity [5]. Histologically, MBs may arise from cells of the inferior rhomboid fossa of the brainstem, granule cell precursors in the external granular layer of the cerebellum, or unipolar brush cells of the superior rhomboidal fossa and cells from glutamatergic nuclei in the cerebellar cortex. Molecular genetic studies using transcriptome analysis and gene methylation profiling have found that these cells differ in their protein and gene expression, leading to the identification of four molecular subtypes: WNT (inferior), SHH (granule cell precursor), and groups 3 and 4 formed from cells of the superior rhomboidal fossa, and cells of the glutamatergic nuclei of the cerebellum [6,7]. Each subgroup has different genetic drivers, clinical signs, and prognoses. The WNT subtype of MB is observed in 10% of patients who have the best prognosis. Their survival rate reaches 90% [8]. Tumors classified as the WNT subtype contain mutations in the WNT pathway, including WNTα, WNT β, CTNNB1, deletion of chromosome 6, and nuclear staining for β-catenin [9,10]. The SHH subtype occurs in 30% of MB patients, including young children (<3 years) and adults (>16 years) [10,11]. It is characterized by activation or mutations in the SHH signaling pathway, such as PTCH1, SMO, and SUFU, as well as amplification of *GLI1* and *GLI2*. Patients with tumors from this subgroup have an intermediate prognosis. Cytogenetic analysis of tumor cells from young children reveals deletions of chromosome 10q and amplification of the *MYCN* gene. In adult MB patients, deletions of chromosomes 10, 2, and 17 and gain of 17q and/or amplification of the *GLI2* gene are observed, which is associated with a poorer prognosis than in children [11]. Group 3 MB patients have worse clinical prognosis, with a 5-year survival rate ranging from 39% to 58%, depending on the patient’s age. Metastasis is recorded in 50% of these cases [12]. Cytogenetic markers in this patient group include an isochromosome i17q in 40–50% of cases; deletions of chromosomes 8 and 10q; and gains of 1q, 7, and 18. *MYCC* and *MYCN* amplification, *SMARCA4* mutations, and *GABRA5* gene overexpression are also present [12]. MB patients in group 4 account for 35–40% of all MBs. Driver mutations in this group include overexpression of *PRDM6* and *GFI1/GFI1B, KDM6A*, *KMT2C*, and *ZMYM3*, as well as amplification of *MYCN, OTX 2*, and *CDK6* [13]. In cytogenetic analysis, the most common (80%) aberration in group 4 tumors is a gain of chromosome 17q, and other mutations include gains of chromosomes 7 and 18q, as well as deletions of chromosomes 8q, 8p, 11p, and X [13].

A lot of these genetic and cytogenetic alterations are used in clinical laboratory diagnostics as biomarkers for stratifying patients into molecular subtypes and clinically favorable and unfavorable risk groups. In molecular groups 3 and 4, which are characterized by the worst clinical prognosis, the most common cytogenetic changes include amplification of the *MYCC* (8q24.12-q24.13) and *MYCN* (2p24) genes, as well as deletion or gain in isochromosome i(17)(10) [14,15]. Amplification of the *MYCC* oncogene was adopted by the International Society for Pediatric Oncology and the Primitive Neuroectodermal Tumour Committee of the European Society of Oncology (SIOP-Europe-PNET) as an official molecular stratification factor for MB in children [16]. For MB, a relationship has been established between the risk of patient mortality and the number of *MYCC* and *MYCN* oncogene copies. The probability of death increases 3.5-fold with an increase of 10 *MYCC* copies and 1.7-fold with each additional *MYCN* copy [17]. On the other hand, the frequency of *MYCC* and *MYCN* amplification in isochromosome 17 (i(17)(q10)) varies in different ethnic populations. For example, in the USA, the frequency of amplification of *MYCC* is 5.2% (4 out of 77 tumors) [18]; in Europe, the frequency for *MYCC* is 3–6% [19]; and in India, it reaches 11% (7 out of 62 cases) [20]. The frequency of i(17)(q10) in MB patients from the USA occurred was 14% (5/35) [21]. Kool et al. conducted an international study involving 550 patients with MB, in which the frequencies of 17p deletions and 17q gains in group 3 were 42% and 62%, respectively, and 73% in group 4 [22]. At the same time, the frequencies of *MYCC, MYCN* amplification, 17q gains and 17p deletions, and i(17)(q10), in the Russian population remain unknown. In addition, although the impact of *MYCC* and *MYCN* amplifications, 17q gains, and 17p deletions on the OS of MB patients has been proven, it is still necessary to explore this relationship with the frequencies of *MYCC, MYCN*, and i(17)(q10) in the context of the Russian population.

**Objective:** The aim is to study the frequency of *MYCC* and *MYCN* amplification, 17q gains, and 17p deletion and determine their prognostic value in Russian MB patients.

## 2. Materials and Methods

### 2.1. Clinical Characteristics of MB Patients

This study was conducted on MB cells obtained from biopsies of 18 patients (12 boys and 6 girls), aged between 3 months and 17 years (median age 6.5 years), who were treated in the children’s neurosurgery department of the Almazov National Medical Research Centre, Ministry of Health, Saint Petersburg, Russia, between 2019 and 2024.

### 2.2. Preparation of Tumor Smears

To conduct this study, smear-imprints of different tumor sections were prepared. Different fragments of the same neoplasm were applied to defatted slides several times so that the cells remained on them. The quality of the material was checked under a light microscope with a magnification of ×100 times. The slides were then dried and left for 24 h at room temperature. They were then fixed in 400 µL of Carnoy’s solution (3:1 methanol/acetic acid, Sigma-Aldrich, St. Louis, MO, USA) overnight [23].

### 2.3. Determination of Cytogenetic Aberrations by FISH Analysis

#### 2.3.1. Preparation of Samples for FISH Staining

The slides treated with fixative were dried for 2 h at room temperature and then transferred to a thermostat at 56 °C for 2–3 h. After cooling to room temperature, the area with the greatest accumulation of cells was identified under a light microscope. The slide was then treated with RNase (10 µg/mL for 15 min at 37 °C) and proteinase K (also 10 µg/mL for another 30 min at the same temperature), followed by two washes in distilled water and drying at room temperature. Following this treatment, the slides were immersed in sodium citrate buffer (0.6 M chloride and 0.06 M sodium citrate at pH 7.2 from Sigma-Aldrich, USA) for 30 min at 38 °C; then, dehydrated using successive concentrations of alcohol (70%, 80%, and 96%) for 2 min each; and finally, dried at ambient temperature for 15–20 min.

#### 2.3.2. Staining of Interphase Nuclei by FISH

DNA probes (7 μL Vysis LSI/WSP buffer, 2 μL water, and 1 μL DNA probe) were applied to slides with cell samples and covered with a coverslip (22 × 22 nm). To determine the amplification of *MYC* (*MYCC* and *MYCN* genes) (copy count > 40–50 copies), we used 0.5 μL locus-specific Vysis LSI DNA probes: *MYCC* (8q24.12-q24.3) and *MYCN* (2p24). These DNA probes were labeled with Spectrum Orange fluorescence (Abbott Molecular, Des Plaines, IL USA). We also used cen17/p53 Dual Color chromosome 17 centromeric probes (Abbott Molecular, USA) to visualize gains or deletions of the *TP53* gene on chromosome 17. After that, we performed DNA denaturation at 73 °C for 5 min, and then, hybridization was carried out in a humidified atmosphere at 37 °C for 16–18 h in a hybridizer (Dako, Carpinteria, CA, USA). Slides were immersed in 0.4× sodium citrate buffer saline solution with 0.3% nonylphenoxypolyethoxyethanol NP40 detergent (Thermo Scientific, Waltham, MA, USA) and heated to 73 °C for 2 min. Slides were then transferred to a sodium citrate-buffered saline solution (0.6 M chloride, 0.06 M citrate, Sigma-Aldrich, USA) containing 0.1% NP-40 for 10 min. Samples were then dehydrated in a series of increasing alcohol strengths (70%, 80%, and 96%) and dried at room temperature for 15–20 min in the dark. Finally, a working solution of DAPI (1.5 μg/mL) was applied to the dried slides and left to stain for 10–15 min, after which a cover glass was placed over the stained area [23,24]. The preparations were analyzed using a Leica DMLB fluorescence microscope (Germany), with filters for DAPI, Texas Red, and FITC, at a total magnification of 1000 (100× oil immersion objective, 10× eyepiece). At least 200 cells per field were counted, and their number was recorded for each set of oncogenes. A total of 24 slides were analyzed, containing 4800 cells. The samples were registered according to the International Nomenclature for Human Cytogenetics (ISCN) 2024 [25].

### 2.4. Statistical Analysis

The results were presented as the arithmetic mean plus/minus the standard error of the mean for the sample (M ± m). One-way ANOVA and the F-test (Fisher) were used to compare the two groups. The differences were considered reliable at a significance level of *p* < 0.05 [26]. Descriptive statistics, ANOVA tests, and OS analyses were performed using GraphPad Prism version 8.01 (21 September 2020) from San Diego, CA, USA.

## 3. Results

**Figure 1:**
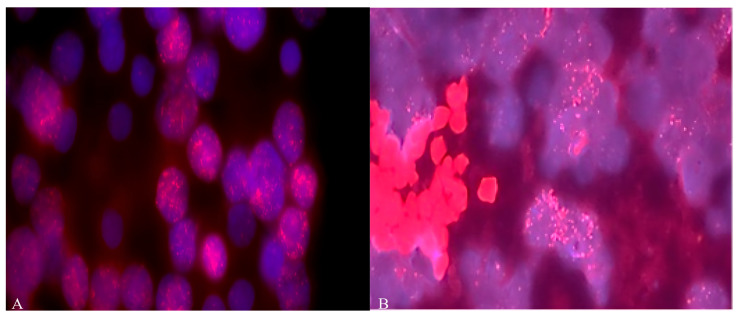
(**A**) Amplification of *MYCN* and (**B**) *MYCC* (copy number more than 60) oncogenes in MB cells from Russian patients. Objective magnification ×100. Blue color indicates DAPI nuclei, red dots indicate staining with *MYCC* (8q24.12-q24.3) and *MYCN (2p24)* probes.

For 77.7% (14/18) of the patients, the classic MB type was found. In 16.7% (3/18), the desmoplastic type was detected, and in 5.6%, a desmoplasic-nodular neoplasm was found [27]. As shown in Figure 1 and Table 1, diffuse amplification of the *MYCN* and *MYCC* genes was detected in more than 22% (4 out of 18) of MB samples from Russian patients, with more than 40–50 copies.

**Figure 2:**
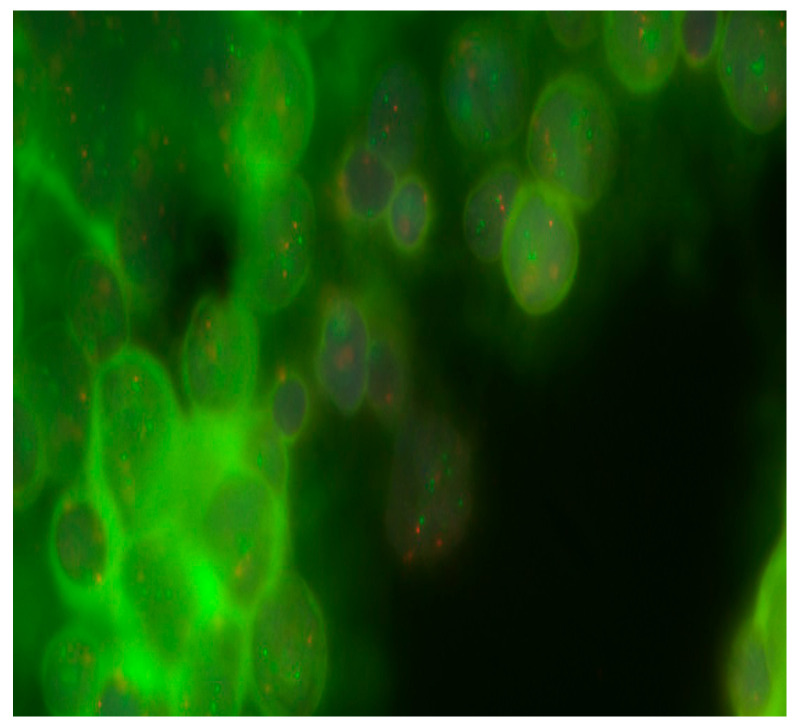
Detection of 17q gain in MB cells from Russian patients. Objective magnification ×100. Green dots are cen17/p53 Dual Color chromosome 17 centromeric probes.

Aberrations in the 17th chromosome were detected in 58.3% (7/13) of patients. Deletions in 17p were detected in 23.1% (3/13), and 17q gains were found in 46.2% (6/13). Table 1 and Figure 2 show that in one case, the presence of i(17)(q10) was identified.

We did not find statistically significant differences between the presence of the 17q and 17p chromosome abbreviations and clinical symptoms in MB patients (Table 2).

**Figure 3:**
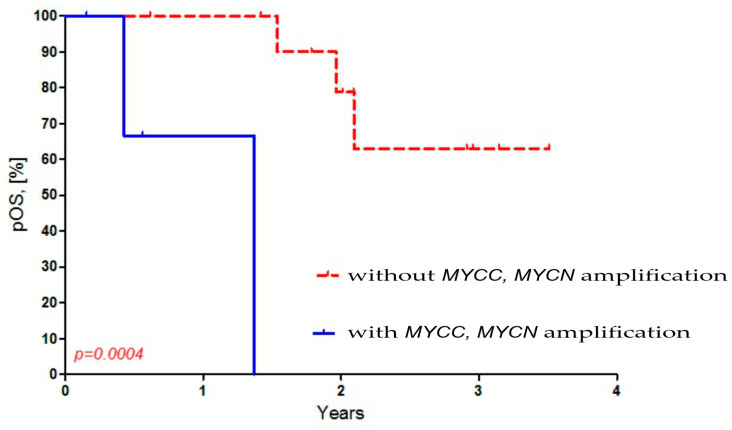
Dependence of overall survival of Russian patients on *MYCC* and *MYCN* amplification in MB cells.

Then, we compared the presence of *MYCC* and *MYCN* amplification in MB cells to the overall survival (OS) in Russian patients. We observed a worse OS in Russian MB patients with both *MYCC* and amplification (*p* = 0.0004). The OS for Russian MB patients who had amplification was 0%, while it was 68% for those without amplification (Figure 3).

**Figure 4:**
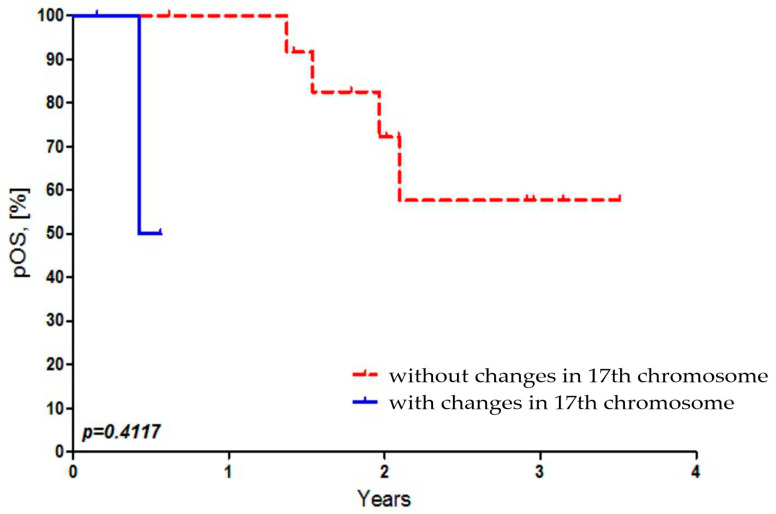
Dependence of overall survival of Russian patients on the presence of 17p and 17q abbreviations in MB cells.

There was no statistically significant dependence found between OS of Russian patients and the presence of 17p and 17q abbreviations in MB cells (Figure 4).

## 4. Discussion

Our results revealed *MYCC* and *MYCN* amplification in 22.2% of Russian MB patients of Slavic origin, which was associated with low OS. Figure 1 and Figure 3 show this association, as does Table 1. Pfister et al. demonstrated that the amplification of *MYCN* and *MYCC* is detected in 4% (3/75) and 6% (5/75), respectively, of German MB cases and is associated with high tumor malignancy and a low (13%) five-year OS for patients. The absence of amplification in these cases is associated with a high (73.0%) five-year survival rate for the patients [28]. In contrast, Moreno et al., using FISH, found *MYCN* amplification in 3.2% (3/92) of SHH subtype MBs in Brazilian patients. The authors also successfully used an nCounter analysis to stratify MBs into molecular subgroups and predict *MYCN* amplification [29]. Aldosari et al. detected *MYCN* amplification by FISH in 5.2% (4/77) of MBs from US patients [18]. A recent study conducted by Schwalbe et al. on 1600 patients with MB characterized the clinical and molecular features of the disease, including amplification of *MYCC* and *MYCN* oncogenes [19]. The amplification frequencies were 4% (64/1600) for the *MYCC* oncogene and 6% (95/1, 600) for the *MYCN* oncogene. The third molecular subtype, represented by *MYC*-amplified SHH-MB, was obtained from patients aged over three. These patients often had metastases, associated with a five-year progression-free survival rate of 11% [19]. There is only one publication on the Internet by Ryzhova et al. (2019), in which the authors used FISH probes to amplify *MYCC* in four Russian patients with MB [30]. However, in this publication, the authors used only *MYCC* probes, not *MYCN* or 17p or 17q. The number of patients was too small to estimate the frequency of amplification of these oncogenes among the Russian population. Therefore, our study is the first to illustrate the frequency of *MYCC* and *MYCN* amplification among MB patients in Russia. In addition, our findings confirm that the frequency of both *MYCC* and *MYCN* amplification in Russian patients with MB is associated with worse OS.

Our data coincide with the results of Pfister et al. (2019) [29], who established the presence of deletions in the 17th chromosome in 53% (42/80) of patients. We did not find an association between the deletion of 17p or gain of 17q and OS in Russian MB patients (Figure 4). In contrast, Park et al. (2020) established this association between the deletion of 17p and poor OS among 30 MB patients [31]. However, this association was not confirmed in independent data cohorts (*n* = 100). The authors suggest that this discrepancy may be due to the interaction between loss of tumor suppressor genes (chromosome 17p) and high expression of *MYCC* or *MYCN* oncogenes. Stratifying patients based on these two factors revealed different survival rates between subgroups: the 5-year OS probability ranged from 19% to 81%. Park et al. showed that age is a significant prognostic factor, after adjusting for chromosome 17, *MYCC* status, and *MYCN* expression. The reduction in OS at less than 3 years is more significant in patients with high expression of either *MYCC, MYCN*, or 17p del [31]. The same authors in a later study indicate that poor OS in the third subgroup of MB is associated with high expression of *MYC* and increased expression of genes such as *PDGFRA, IGF1R*, and *FGF2*, as well as the i(17)(q10), PI3K/AKT, and MAPK/ERK pathways [32]. It is possible that the differences between our data and those of Park et al. on chromosome 17 [31] may be due to the small number of patients (*n* = 6) in whom these changes were detected (Table 1). Interestingly, in a recent study (2024), Vriend and Liu identified 967 genes associated with the OS of MB patients using a gene expression dataset and a Cox proportional hazards regression model [33]. These genes were localized to chromosomes 6 and 17 in MB cells. They include the oncogene high-mobility group protein HMG-I/HMG-Y (HMGA1) on chromosome 6, whose high expression is associated with poor OS in patients. Genes on chromosomes 17p del and 17q gain that are associated with telomere lengthening are linked to low OS in MB patients [33].

FISH analysis is the “gold standard” in cytogenetics because it allows for quantitative detection of aberrant changes with high sensitivity and specificity, even in very small numbers of cells among cells with a normal karyotype, using fresh and frozen tissue, cytological preparations, and formalin-fixed paraffin embedded tissue. In this respect, validation of this method is generally not required [23,34].

The present pilot study has several limitations. First, it has a small sample size. It is possible that, with an increased number of MB patients, the frequency of *MYCC* and *MYCN* amplifications, and 17p and 17q abbreviations in MB will change during clinical trials. Second, the retrospective design of this study may lead to changes in OS for patients. Third, MB is a heterogeneous tumor that includes four or more cellular and molecular subtypes. These characteristics affect OS patients, which, given their small sample, may affect the frequency of *MYCC* and *MYCN* amplification, as well as chromosome 17 abnormalities, and a different life expectancy. Also, FISH analysis does not allow for the detection of large genomic changes. It is characterized by signal attenuation, a limited number of commercial probes, and cytological artifacts [34].

## 5. Conclusions

The obtained results of this pilot study indicate for the first time a high frequency of detection of *MYCC* and *MYCN* amplification, and *17p* del/*17q* gain abbreviations in a small cohort of MB patients from Russia. However, an increase in the number of MB patients due to multicenter collaboration will allow us to correct and verify the frequency of cytogenetic abnormalities in Russian patients with MB. In this group of people from Russia, the association between *MYCC, MYCN*, and OS of patients was confirmed. Detection of *MYCC* and *MYCN* amplification by i-FISH analysis allows for the classification of patients’ tumors into three or four molecular subtypes associated with an unfavorable clinical prognosis. This allows for the correction of treatment protocols (drugs, regimens, and doses) before its start and thereby increases therapy effectiveness.

## Figures and Tables

**Table 1:** Amplification of *MYCC* and *MYCN* oncogenes, and 17p del and 17q gain aberrations in MB cells from Russian patients.

Patient No.	*MYCN* Amp	*MYCC* Amp	17p Del	17q Gain
1	-	-	+	+
2	-	-	+	-
3	-	-	-	-
4	-	-	-	+
5	-	-	n.d.	n.d.
6	-	-	-	+
7	amp	-	-	+
8	-	-	-	_
9	-	-	-	+
10	-	-	n.d.	n.d.
11	-	-	-	-
12	-	-	n.d.	-
13	amp	-	n.d.	n.d.
14	-	amp	+	+
15	-	-	n.d.	n.d.
16	-	-	n.d.	n.d.
17	-	amp	-	-
18	-	-	n.d.	-

Note: Amp—amplification; presence “+” or absence “-” of 17p del and 17q gain, respectively; Chr.17 was not available (n.d.).

**Table 2:** Dependence between 17q and 17p chromosome abbreviations and the clinical signs of patients with MB from Russia (*n* = 13).

Clinical Criteria	Changes in the 17th Chromosome	No Changes in the 17th Chromosome	*p* Value
Patients	7	6	
Gender			*0.1643*
boys	6	3	
girls	1	3	
Age			
younger than 3 years	2	1	0.6115
older than 3 years	5	5	
Histological type			
desmoplastic	1	1	0.9056
classic	6	5	0.9056
anaplastic	-		
Metastasis			
yes	3	1	0.3077
no	4	5	0.3077
Tumor resection:			
total	2	2	0.8529
subtotal	5	4	0.8529

## Data Availability

Experimental data can be provided by the authors upon request.
